# The Role of Cervical and Ocular Vestibular Evoked Myogenic Potentials in the Assessment of Patients with Vestibular Schwannomas

**DOI:** 10.1371/journal.pone.0105026

**Published:** 2014-08-19

**Authors:** Elodie Chiarovano, Cynthia Darlington, Pierre-Paul Vidal, Georges Lamas, Catherine de Waele

**Affiliations:** 1 CESEM – CNRS UMR 8194 – Université Paris Descartes, Centre Universitaire des Saints-Pères, Paris, France; 2 Department of Pharmacology and Toxicology, University of Otago Medical School, Dunedin, New Zealand; 3 ENT Department – Salpêtrière Hospital, Paris, France; University of Iowa, United States of America

## Abstract

**Objectives:**

To investigate the clinical utility of VEMPs in patients suffering from unilateral vestibular schwannoma (VS) and to determine the optimal stimulation parameter (air conducted sound, bone conducted vibration) for evaluating the function of the vestibular nerve.

**Methods:**

Data were obtained in 63 patients with non-operated VS, and 20 patients operated on VS. Vestibular function was assessed by caloric, cervical and ocular VEMP testing. 37/63 patients with conclusive ACS ocular VEMPs responses were studied separately.

**Results:**

In the 63 non-operated VS patients, cVEMPs were abnormal in 65.1% of patients in response to AC STB and in 49.2% of patients to AC clicks. In the 37/63 patients with positive responses from the unaffected side, oVEMPs were abnormal in 75.7% of patients with ACS, in 67.6% with AFz and in 56.8% with mastoid BCV stimulation. In 16% of the patients, VEMPs were the only abnormal test (normal caloric and normal hearing). Among the 26 patients who did not show oVEMP responses on either side with ACS, oVEMPs responses could be obtained with AFz (50%) and with mastoid stimulation (89%).

**Conclusions:**

The VEMP test demonstrated significant clinical value as it yielded the only abnormal test results in some patients suffering from a unilateral vestibular schwannoma. For oVEMPs, we suggest that ACS stimulation should be the initial test. In patients who responded to ACS and who had normal responses, BCV was not required. In patients with abnormal responses on the affected side using ACS, BCV at AFz should be used to confirm abnormal function of the superior vestibular nerve. In patients who exhibited no responses on either side to ACS, BCV was the only approach allowing assessment of the function of the superior vestibular nerve. We favor using AFz stimulation first because it is easier to perform in clinical practice than mastoid stimulation.

## Introduction

Vestibular schwannoma (VS) is a benign tumor that develops from Schwann cells of the vestibular nerve. The tumor arises within the internal auditory canal (IC) and grows into the cerebello-pontine angle (CPA), resulting in a specific pattern of symptom development. The vestibular nerve is involved in most cases of VS [1] and patients show various patterns of vestibular dysfunction.

Several tests are currently available to investigate vestibular function in patients with suspected vestibular schwannoma. The caloric test and the head impulse test [2] are useful for determining the function of the horizontal semi-circular canal. Air-conducted sound (ACS) and bone-conducted vibration (BCV) elicit cervical and ocular vestibular-evoked myogenic potentials (VEMPs) that are now widely used to assess otolith function [3]–[4].

**Figure 1:**
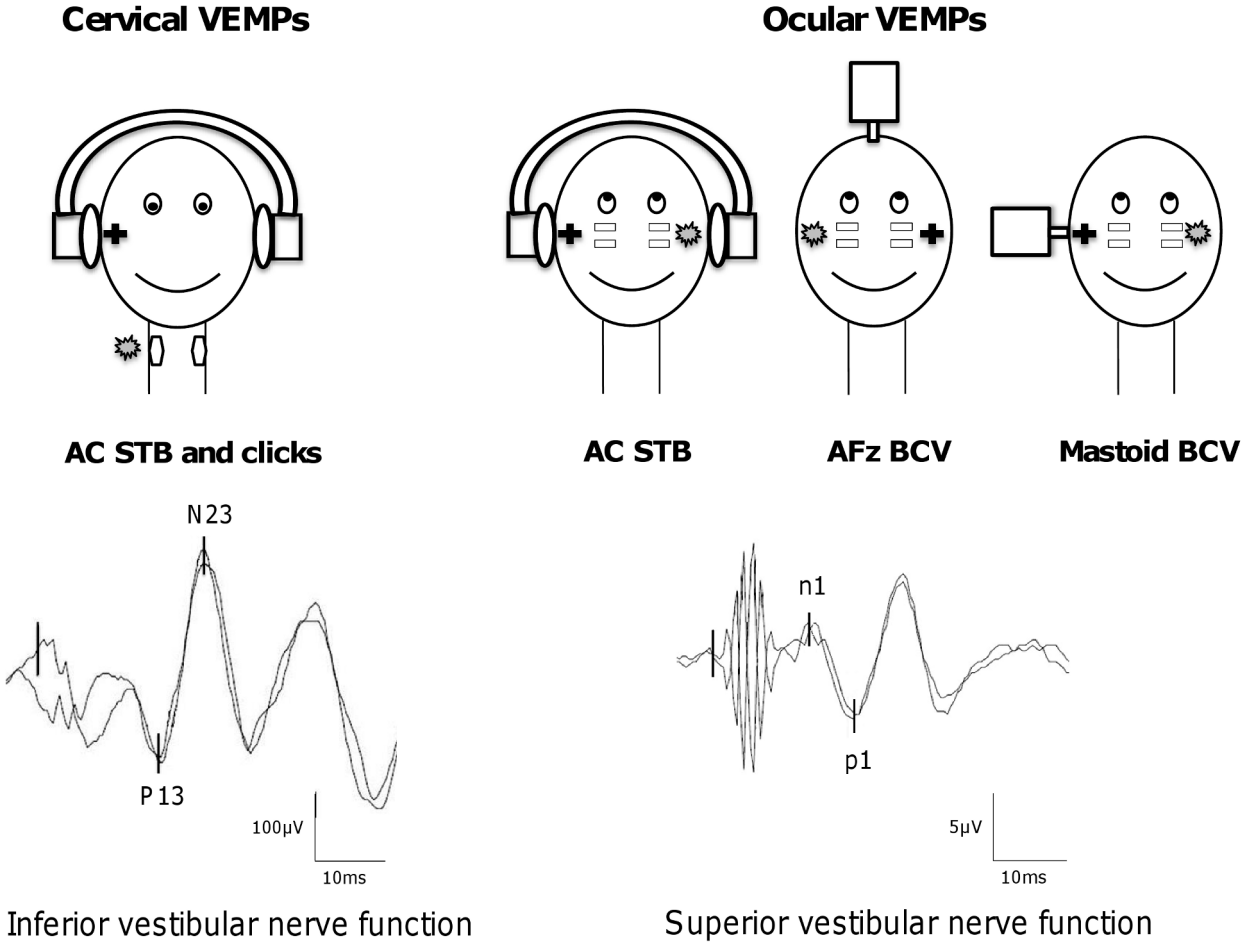
Presentation of the five VEMP tests. Cervical VEMPs (mostly saccular function) are induced by air-conducted sounds through headphones using either 500 Hz short-tone burts (AC STB) or high level clicks. Ocular VEMPs (mostly utricular function) are evoked by either AC STB delivered through headphones, or bone-conducted vibration through a mini-shaker applied at AFz (AFz BCV) or at the mastoid (mastoid BCV). The sign + indicates the stimulated ear and the grey banner corresponds to the site of recording. Cervical VEMPs are composed of two early waves: P13 and N23 (scale bar: 100 µV). Ocular VEMPs are composed of two early waves, n1 and p1, with smaller peak-to-peak amplitudes (scale bar: 5 µV).

Cervical VEMPs (cVEMPs) result from the activation of the uncrossed vestibulo-collic reflex [5] and appear to originate predominantly from the inferior vestibular nerve and the saccular macula. cVEMPs may be evoked by ACS, either short-tone bursts (STB) or high level clicks through headphones (Figure 1). Ocular VEMPs (oVEMPs) are a manifestation of the crossed vestibulo-ocular reflex [6]–[7]. The otolithic input to the contralateral oculomotor muscles appears to originate predominantly from utricular macula via the superior vestibular nerve. oVEMPs may be elicited by AC STB through headphones or by BCV at AFz and at the mastoid process via a mini-shaker (Figure 1). ACS and BCV activate both saccular and utricular afferents, although the patterns of activation for ACS and BCV are not identical [3].

At least five VEMP tests are currently available for use in clinical practice to evaluate otolithic function [8] (Figure 1).

Abnormal VEMPs in VS patients have been reported. However, the vestibular function of patients has not previously been studied using all five VEMP tests. There is not consensus about the optimal testing approach to such patients.

Our aims of this study were threefold: to investigate the clinical utility of VEMP testing to assess the function of the superior and inferior vestibular nerves; to determine the optimal mode of stimulation for detecting abnormal function of the inferior and/or the superior vestibular nerve; and to determine in which order ACS and BCV should to be used in routine clinical practice to best assess the function of the superior vestibular nerve and predominantly of utriculo-ocular pathways.

## Materials and Methods

The protocol was approved by the CPP ile de France VI Pitie Salpetriere in accordance with the Declaration of Helsinki. All patients gave their written informed consent.

### 2.1. Subjects

This retrospective study included 122 subjects made up of a control group (n = 39) and a group of patients with unilateral VS including patients who had undergone surgery (n = 20) and who had not undergone surgery (n = 63).

**The control group** consisted of 39 normal subjects (22 women and 17 men; mean age 41.3±13.3 years; age range 20–72 years) with no hearing loss and no bilateral or unilateral vestibular disorders.

**The patient groups** consisted of 20 patients with complete unilateral vestibular loss after surgery for VS (12 women and 8 men; aged 29 to 79 years; mean age 49.5±12.6 years) and 63 unoperated patients diagnosed with unilateral VS (39 women and 24 men; aged 27 to 82 years; mean age 56.1±13.5 years). All patients were diagnosed after cerebral MRI.

Thirty-seven of the 63 unoperated patients could be analyzed with all oto-neurologic tests. They showed oVEMPs responses to AC STB from the intact side (responder patients), and so the comparison between the three modes of oVEMP stimulation was possible. The other 26 patients exhibited no oVEMP responses to ACS stimulation on either side (non-responder: NR patients). They were only analyzed for oVEMP responses to compare AFz and mastoid BCV.

### 2.2. VEMP testing

VEMPs were recorded with a Nicolet Viking 4 apparatus (Nicolet Biomedical Inc., Madison, WI) with a 4-channel averaging capacity, as previously described [9]–[11].

The clicks (0.1 ms rarefactive square waves of 105 dB nHL) and STB (500 Hz, 102 dB nHL, 128 dB SPL, rise/fall time 2 ms, plateau time 2 ms) were presented through calibrated TDH39 headphones. BCV stimuli were 500 Hz STB (rise/fall time = 2 ms and plateau time = 2 ms). They were delivered by a hand-held Bruel and Kjaer (Naerum, Denmark) Mini-Shaker 4810, fitted with a short bolt (2 cm long, M4) terminating in a bakelite cap 1.5 cm in diameter, which was the contact point for the stimulator on the subject. The Mini-Shaker weighs approximately 1 kg and the weight of the shaker was used to standardize the force applied to the subjects. The Mini-Shaker was calibrated using a sound level meter (Bruel and Kjaer 2250, calibrated to read 0 dB at 1 µV) and an artificial mastoid (Bruel and Kjaer 4930). The voltage drive peak to peak used for 500 Hz STB was 12 V, corresponding to a 135 dB FL.

cVEMPs were recorded from the sternocleidomastoideus (SCM) muscles ipsilateral to the stimulated ear in response to AC STB and click stimuli. Patients lay supine on a bed and were asked to lift their head off the pillow and orient it contralaterally to the ear tested to activate maximally the SCM muscle ipsilateral to the stimulation. EMG activity of the SCM was monitored on a display to ensure that sufficient muscle activation was maintained (>150 µV). Latencies of the two early waves (P13 and N23) of the cVEMPs were measured in ms, and the peak-to-peak amplitude between P13 and N23 waves was measured in µV.

oVEMPs were recorded from the extraocular muscles contralateral to the stimulated ear in response to AC STB, to BCV at AFz (to the midline forehead at the hairline, the skull location identified as Fz or AFz) and at the mastoid (just behind the pinna of the ear on the mastoid process with the Mini-Shaker held perpendicular to the skin surface). Patients lay supine on a bed and were asked to direct their gaze at a target located 1 m away at an elevation of 25 degrees. The active self-adhesive electrode was placed on the orbital margin, 0.5 cm below the lower eyelid and referred to a parallel electrode below it (approximately 2 cm below the lower eyelid). We measured the peak latencies in ms and the peak-to-peak amplitude in µV of the two early waves (n1 and p1). If the peak-to-peak n1-p1 amplitude was smaller than 2 µV, the response was considered as absent.

The percentage of VEMP asymmetry in patients with unilateral vestibular lesions was measured by calculating the evoked potential ratio (EPr) as follows [12]: EPr = 100*(Al – As)/(Al + As), where Al is the larger P13-N23 or n1-p1 peak-to-peak amplitude, As is the smaller P13-N23 or n1-p1 peak-to-peak amplitude.

Patients with a positive response from the intact side were defined as responder subjects. Patients with no response on either side were considered as non-responders (NR). In responder subjects, the response was defined as normal if the EPr was below the threshold value and abnormal if EPr was above the threshold. This threshold was calculated as the mean EPr for the control group +2SD for the five VEMP tests.

### 2.3. Caloric testing

Caloric tests were performed using closed loop sequential bithermal irrigation with water at 30 and 44°C and video-nystagmography. Percent of canal paresis (CP) was calculated using Jongkees' formula [13]: CP = 100*((UW+UC) – (AW+AC))/(UW+UC+AW+AC), where UW, UC, AW and AC are velocity of the induced ocular nystagmus obtained on the unaffected and affected sides, with warm and cold water, respectively. A value of CP greater than 20% was regarded as an abnormal decrease on the affected side.

### 2.4. Audiometric tests

Tympanometry and stapedial reflex were carefully evaluated to exclude patients with a conductive (even slight) hearing loss to avoid misinterpretation of ACS oVEMPs. The mean pure-tone threshold (PTA) for tones at 500 Hz, 1 kHz and 2 kHz was used as an indicator of hearing loss.

### 2.5. Statistical analysis

Statistical analyses were performed using SAS 9.3 statistical software. For comparisons of numerical data, we used a non-parametric Wilcoxon test or a t-test according to the normality distribution of the data. For comparisons of the distribution (percentage), we used an exact Fisher test or a χ^2^ test according to the expected effects. A difference of p<0.05 was considered as significant.

## Results

### 3.1. Healthy subjects

All subjects in the healthy control group (n = 39) showed a positive response to all the five VEMP tests.

The mean cVEMP peak-to-peak amplitude in response to AC STB was significantly greater than that in response to AC clicks (paired t-test, Table 1). The mean P13 and N23 latencies were significantly shorter for AC click cVEMPs than for AC STB cVEMPs (paired t-test, Table 2).

**Table 1:** Means (±SD) peak-to-peak amplitude of cVEMPs induced by AC STB and clicks, and mean (±SD) peak-to-peak amplitude of oVEMPs induced by AC STB and BCV (AFz and mastoid), in healthy subjects, 63 patients with non-operated VS (affected and intact side) and 20 patients after surgery for VS (intact side).

VEMP tests	Stimulation	Healthy subjects	Non-operated VS affected side	Non-operated VS intact side	Operated VS intact side
cVEMP amplitudes (µV)	AC STB P13/N23	324±158	93±136*	265±158 n = 60	228±122
	AC clicks P13/N23	138±63	36±68*	141±87 n = 41	109±48
oVEMP amplitudes (µV)	AC STB n1/p1	6.6±3.5	2.1±3.8*	7.0±4.0 n = 37	5.3±4.8
	AFz BCV n1/p1	10.1±6.2	2.9±4.7*	9.8±7.6 n = 50	8.1±3.3
	Mastoid BCV n1/p1	16.2±9.9	6.0±9.2*	13.8±10.1 n = 60	10.8±5.3

*Significantly different to healthy subjects (*t*-test).

VS: vestibular schwanomma.

VEMPs: vestibular evoked myogenic potentials.

cVEMPs: cervical VEMPs.

oVEMPs: ocular VEMPs.

AC: air-conducted.

BCV: bone-conducted vibration.

STB: short-tone bursts.

**Table 2:** Mean (±SD) latencies of cVEMP induced by STB and clicks (P13 and N23) and of oVEMPs induced by AC STB and BCV (AFz and mastoid; n1 and p1), in healthy subjects, 63 patients with non-operated VS (affected and intact side) and 20 patients after surgery for VS (intact side).

VEMP tests	Stimulation	Healthy subjects	Non-operated VS affected side	Non-operated VS intact side	Operated VS intact side
cVEMP latencies (ms)	AC STB P13	14.8±1.0	14.7±1.4 n = 29	14.8±1.3 n = 60	14.6±0.9
	AC STB N23	21.6±1.3	21.5±2.3	21.9±1.8	21.2±1.2
	AC clicks P13	11.9±1.1	12.7±1.9 n = 12	12.1±1.7 n = 41	12.1±1.2
	AC clicks N23	18.3±1.6	18.7±3.1	18.1±2.1	18.2±1.2
oVEMP latencies (ms)	AC STB n1	11.2±0.8	11.3±0.7 n = 12	11.2±0.7 n = 37	11.6±0.6
	AC STB p1	15.3±1.0	14.9±1.5	15.1±1.2	16.2±1.3
	AFz BCV n1	11.2±0.8	11.4±0.8 n = 17	11.2±0.8 n = 50	11.1±0.7
	AFz BCV p1	15.2±1.1	14.8±1.7	14.9±0.9	15.0±0.8
	Mastoid BCV n1	10.9±0.7	11.5±1.4 n = 25	11.15±1.3 n = 60	10.8±0.5
	Mastoid BCV p1	15.3±1.0	15.5±2.4	15.3±1.7	14.6±0.6

Note that no significant differences were detected in the latencies of P13, N23 and n1-p1 between healthy subjects and VS patients.

The mean oVEMPs peak-to-peak amplitude was lower in response to AC STB than in response to AFz BCV. Similarly, the response to AFz BCV was lower than the response to mastoid BCV (paired t-test, Table 1). There was no significant difference between the mean latencies of n1 and p1 between AC STB, AFz BCV and mastoid BCV stimulations (paired t-test, Table 2).

The EPr of the AC STB cVEMPs ranged from 0 to 38.9% (mean ± SD, 15.5±11.4%). On the basis of these findings, we defined **39%** (mean+2SD) as the upper limit of the normal range of the cervical STB EPr. The mean cervical click EPr was 16.0±10.9% and the upper limit of the normal range of the cervical click EPr was thereby defined as **38%**. The mean ocular AC STB EPr was 15.2±10.8% (range from 0 to 35.8%) and the upper limit of the normal range for the ocular AC STB EPr was **37%**; the corresponding upper limits were **32%** for AFz BCV EPr (mean ocular AFz BCV EPr: 14.6±8.3%; range: 1.1 to 31.6%) and **24%** for mastoid BCV EPr (mean ocular mastoid BCV EPr: 10.0±6.6% range 0 to 22.7%).

### 3.2. Patients suffering from a unilateral non-operated VS

This group consisted of 63 patients with unilateral VS (39 women and 24 men, mean age: 56.1±13.5 years). There were 36 CPA and 27 IC VS. Hearing loss and vestibular test results (caloric test, AC STB cVEMPs and oVEMPs) showed that most patients (85%) exhibited at least one abnormal response to the three vestibular tests; however only 33% suffered from PTA hearing loss. Therefore, vestibular dysfunction in our population was more frequent than hearing dysfunction. Caloric test results were abnormal for 45/63 patients (71%; CP = 66.2±26.4%, Table 3).

**Table 3:** Number (%) of non-operated VS patients (n = 63) exhibiting abnormally weak (decreased or abolished) response from the affected side or normal responses to vestibular tests (ACS cVEMPs, AC STB and BCV oVEMPs, and caloric tests).

Non-operated VS (n = 63)		Decreased response from the affected side	Abolished response from the affected side	Normal response	No response on either side
cVEMP tests	AC STB	10 (15.9%)	31 (49.2%)	19 (30.1%)	3 (4.8%)
	AC clicks	2 (3.2%)	29 (46.0%)	10 (15.9%)	22 (34.9%)
oVEMP tests	AC STB	3 (4.7%)	25 (39.7%)	9 (14.3%)	26 (41.2%)
	AFz BCV	4 (6.3%)	33 (52.4%)	13 (20.6%)	13 (20.6%)
	Mastoid BCV	7 (11.1%)	35 (55.5%)	18 (28.5%)	3 (4.8%)
Caloric test		29 (46.0%)	16 (25.4%)	18 (28.6%)	0

Table 3 illustrates, for cVEMPs (STB and clicks) and oVEMPs (ACS, BCV AFz and mastoid), the percentage of normal and abnormal (decreased or abolished) responses in the 63 VS patients, and the percentage of patients NR on each the affected and unaffected sides.

The latencies of P13, N23 cVEMPs and of n1, p1 oVEMPs, from the affected side and from the intact side were not significantly different from those in the control group for all cVEMP and oVEMP stimulations. Thus, the reduced peak-to-peak amplitude of the VEMP response from the affected side was not associated with longer latencies (Table 2).

Note that for two of the five stimulations (AC clicks for cVEMPs and AC STB for oVEMPs), numerous patients were NR (35% and 41%, respectively). One of the aims of this work for oVEMPs was to compare the optimal stimulations between ACS and BCV at AFz and at the mastoid. Consequently, we decided to subdivide the patient group as follows:

A group of 37 patients with positive response from unaffected side to all the three oVEMP stimulations (0% NR).
A group of 26 patients NR to AC STB oVEMPs.

#### Responder patients to AC STB oVEMPs (n = 37)

This group consisted of 37 patients with unilateral VS (21 women and 16 men, mean age: 51±12.1 years). Abnormal responses on the affected side for all of the caloric test, AC STB cVEMPs and oVEMPs, were observed in 51% of the patients (n = 19). Caloric test results were abnormal for 25 patients (67.6%; CP = 65.2±26.2%, Table 4). There were significant associations between caloric test results and both cVEMPs (χ^2^, p = 0.02) and oVEMPs (χ^2^, p<0.001). However, inconsistencies were observed between caloric test results and AC STB cVEMPs (n = 10; 27%) and between caloric test results and AC STB oVEMPs (n = 5; 14%).

**Table 4:** Number (%) of responder non-operated VS patients (n = 37) exhibiting abnormal decreased response, abnormal abolished response from the affected side (EPr = 100%) or normal response to vestibular tests (ACS cVEMPs, AC STB and BCV oVEMPs, and caloric tests).

Non-operated VS (n = 37)		Decreased response from the affected side	Abolished response from the affected side	Normal response	No response on either side
cVEMP tests	AC STB	8 (21.6%)	15 (40.5%)	14 (37.9%)	0
	AC clicks	0	21 (56.8%)	9 (24.2%)	7 (19%)
oVEMP tests	AC STB	3 (8.1%)	25 (67.6%)	9 (24.3%)	0
	AFz BCV	3 (8.1%)	22 (59.5%)	12 (32.4%)	0
	Mastoid BCV	3 (8.1%)	18 (48.7%)	16 (43.2%)	0
Caloric test		17 (45.9%)	8 (21.7%)	12 (32.4%)	0

**Figure 2:**
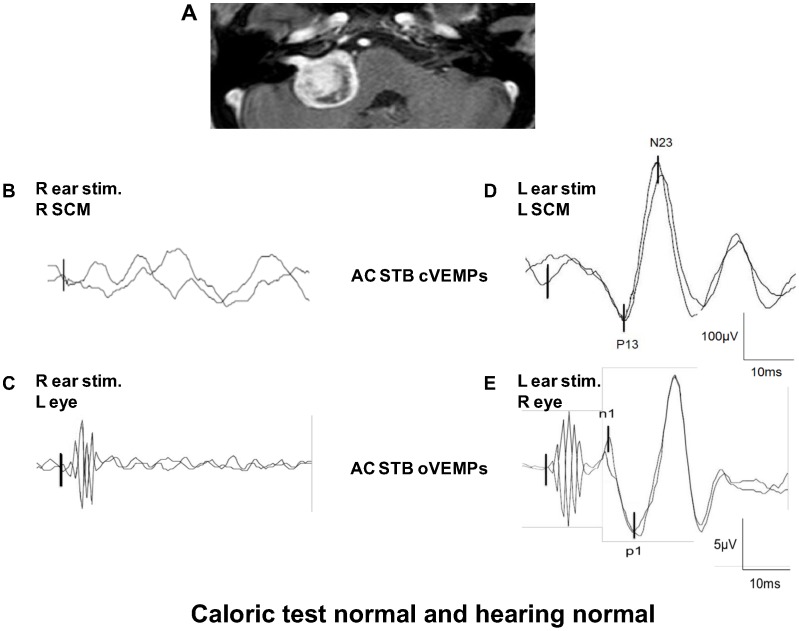
Clinical utility of VEMP test in vestibular schwannoma patients. This patient has a right VS in the cerebello-pontine angle. Despite the size of the VS (MRI in **A**), he had both normal hearing and caloric test results. Patient had superior nerve dysfunction (absence of AC STB cVEMP on the right SCM when the right ear was stimulated: **B**) and inferior nerve dysfunction (abnormal AC STB oVEMPs on the left eye when the right ear was stimulated: **C**). When the left intact ear was stimulated, normal AC STB cVEMPs (**D)** and normal AC STB oVEMPs (**E**) were observed.

In these 37 patients, six patients (16%) suffering from balance problems and vertigo, had normal hearing and normal caloric test results, **but** abnormal cervical and/or ocular VEMP test results (Table 5). All six patients had undergone MRI, due to the abnormal VEMPs, which revealed the VS. This illustrates the possible clinical value of VEMP testing as it can indicate the need for more detailed investigations, for example, MRI centered on the IAC. Figure 2 illustrates the vestibular data obtained in one patient suffering from a right APC VS (patient 5). No cVEMP and oVEMP responses could be obtained from the affected right side, with any mode of stimulation, whereas the other tests (audiometric, caloric) were normal. This patient was operated and the diagnosis of VS was confirmed by histology.

**Table 5:** Results for six VS patients with abnormal cervical and/or ocular VEMPs but normal caloric and hearing tests.

	Location	Age/Sex	Caloric test	Audiometric test	AC STB cVEMPs	AC STB oVEMPs	AFz BCV oVEMPs	Mastoid BCV oVEMPs
Patient 1	CPA	51/M	Normal	Normal	Normal	Abolished	Abolished	Abolished
Patient 2	IC	65/M	Normal	Normal	Normal	Abolished	Abolished	Abolished
Patient 3	CPA	45/M	Normal	Normal	Abolished	Normal	Normal	Normal
Patient 4	IC	37/M	Normal	Normal	Decreased	Abolished	Decreased	Normal
Patient 5	CPA	49/F	Normal	Normal	Abolished	Decreased	Normal	Normal
Patient 6	IC	60/F	Normal	Normal	Abolished	Decreased	Normal	Normal

Patient 5 is illustrated in figure 2.

CPA: cerebello-pontine angle.

IC: intracanalar.

The response was defined as normal if the EPr was below the threshold value and abnormal (abolished or decreased) if EPr was above the threshold.

Table 4 shows the percentages of normal and abnormal responses to the five VEMP tests in the 37 patients with positive oVEMP responses from the affected side.

For AC STB cVEMPs, 23/37 patients (62.1%) showed abnormal responses and 14/37 (37.9%) patients showed normal responses. For AC clicks, 21/37 (56.8%) patients showed abnormal cVEMPs from the affected side; 9/37 (24.2%) showed normal responses. The number of NR increased from 0% for STB to 19% (7/37) for clicks (Table 4). No significant difference was found between the percentages of abnormality of responses to the two types of stimulation (AC STB and AC clicks).

For AC STB oVEMPs, 28/37 (75.7%) patients had abnormal responses. Nine of the 37 (24.3%) patients presented normal oVEMP responses to AC STB stimulation. For AFz BCV oVEMPs, 25/37 (67.6%) patients had abnormal responses and 12/37 (32.4%) had normal oVEMP responses. For mastoid BCV oVEMPs, 21/37 (56.8%) patients had abnormal responses and 16/37 (43.2%) had normal oVEMP responses. No significant difference was found between the percentages of abnormality of responses to the three types of stimulation (AC STB, AFz BCV and mastoid BCV).

#### Non-responder patients to AC STB oVEMPs (n = 26, NR patients to ACS)

This group consisted of 26 patients with unilateral VS (18 women and 8 men, mean age: 63.4±12.2 years). There were 15 CPA and 11 IC VS.

In 26 patients with no response to AC STB oVEMPs on either side, cVEMP response could be obtained in 88% (5 normal, 2 dicreased, 16 abolished and 3 NR) with STB and in 42% with clicks (2 normal, 9 abolished and 15NR).

In 26 patients with no response to AC STB oVEMPs on either side, oVEMPs response could be obtained in 50% with AFz and in 89% with mastoid BCV stimulation.

Abnormal oVEMPs to AFz stimulation were detected in 46% of the patients (n = 12/26) and all these patients had also abnormal oVEMPs to mastoid BCV (n = 12). One patient had normal oVEMPs to AFz and to mastoid BCV stimulation.

Among NR patients for AFz (n = 13), abnormal oVEMPs to mastoid BCV were detected in 69% of patients (n = 9/13) and normal responses were detected in 8% (n = 1). No response was observed on either side in 23% of the patients (n = 3).

### 3.3. VEMPs in patients after surgery for a unilateral VS

This group consisted of 20 subjects who had undergone surgery for a left (6 patients) or a right (14 patients) VS between 7 days and 18 years earlier (mean range 30.05±58.77 months). Only two types of responses were observed: absent responses from the affected side and absent responses from both sides (i.e. NR patients). All the results for these patients are given in table 6. Note that 50% of subjects were NR to ACS oVEMPs whereas only 15% of subjects were NR for AFz BCV and 5% for mastoid BCV stimulation.

**Table 6:** Number (%) of operated VS patients (n = 20) with abnormal responses or no responses on either side to VEMP tests.

Operated VS (n = 20)		Abolished response from the affected side	No response on either side
cVEMP tests	AC STB	18 (90%)	2 (10%)
	AC clicks	14 (70%)	6 (30%)
oVEMP tests	AC STB	10 (50%)	10 (50%)
	AFz BCV	17 (85%)	3 (15%)
	Mastoid BCV	16 (80%)	1 (5%)

Note that for the mastoid BCV stimulation, three patients were not included because of the consequences of the surgery on the mastoid process.

## Discussion

In this study, we analyzed the vestibular profile of vestibular schwannoma (VS) patients. We also investigated which modes of stimulation (ACS versus BCV) were optimal for evaluating the function of the superior vestibular nerve through the analysis of oVEMPs.

In the 63 patients, the overall sensitivity of all the tests performed was 85%. Caloric tests were abnormal in 71.4%, in agreement with previous studies [14]–[15]. More dissociation was observed between cVEMP and caloric test results than oVEMP and caloric test results [16]–[17]. This suggests that AC STB oVEMPs mainly reflect the function of the superior vestibular nerve [18]. The associations between cVEMPs and caloric test results may have been a result of more than 50% of our population showing abnormal results for all the three neuro-otologic tests (caloric, cVEMPs and oVEMPs).

### Clinical utility of VEMPs

In the 37 VS patients analyzed with all our oto-neurologic tests (i.e. in patients with a positive response to AC STB from the unaffected side), abnormal function of the superior vestibular nerve (abnormal oVEMPs: 76%) was observed more frequently than abnormal function of the inferior vestibular nerve (abnormal cVEMPs: 62%). More importantly, in 16% of these patients, only the superior and/or the inferior vestibular nerve were found to be dysfunctional when tested by VEMPs. All these patients had both normal function of the auditory nerve (normal hearing) and normal function of the horizontal canal nerve (as assessed by caloric tests). These data reveal the possible value of VEMP testing for the diagnosis of VS patients and their follow-up [19]–[20]. More importantly, these data modified our clinical practice. VEMPs were done systematically in patients suffering from vertigo or imbalance and an MRI centered on the IAC was requested in the case of isolated abnormal cervical or ocular VEMPs. Finally, cVEMPs and oVEMPs allow evaluation of the effects of treatment on VS patients who undergo stereotactic radiosurgery [21]. The role of the VEMPs in VS is not primarily in diagnosis, although some patients were detected this way. We feel their main role is to assess the function of the superior and inferior vestibular nerves prior to surgery and/or microradiosurgery. The findings are as important as the horizontal and vertical vHIT tests. They are also an important means of following unoperated patients.

For patients in whom ablative treatment is offered, the baseline results of VEMP testing have at least two important applications: first, in surgical patients, as a guide to how much residual vestibular function is present and thus whether patient is likely to require vestibular rehabilitation post treatment and, second, in radiosurgery patients who become unsteady post treatment, to help determine whether this is due to decompensation or to further compromise of vestibular function.

### cVEMPs: STB or clicks stimulation?

Dysfunction of the inferior vestibular nerve was detected in 65% of the 63 VS patients and in 62% of the 37 VS patients with positive response from the unaffected side in response to AC STB stimulation. The rate of abnormality was not significantly different between the two groups. For click stimulation, 49% of the group of 63 VS patients and 56% of the group of 37 VS patients exhibited abnormal function of the inferior vestibular nerve. This difference may be because more patients in the 63 VS patient group (35%) than the 37 VS patient group (19%) were NR to clicks.

AC STB should be performed first because this stimulation is more effective than clicks for testing sacculo-spinal pathways [22]–[24]. As previously shown, both the energy and frequency of the stimulus modify the amplitude of cVEMPs. It should also be recalled that, due to frequency tuning of the vestibular system [24], a 500 Hz STB sine wave was considered to be the most effective waveform to stimulate the inferior vestibular nerve.

Nevertheless, clicks are useful in a second step to detect minor dysfunction of the inferior vestibular nerve: patients with normal STB cVEMP responses may exhibit abnormal responses to clicks (as was the case for three of our patients).

### oVEMPs: Which mode of stimulation should be used: ACS alone or ACS and BCV together?

The percentages of the group of 63 patients giving abnormal results were 44% for AC STB, 59% for AFz BCV and 67% for mastoid BCV. However, our findings for the group of 37 responder patients were different: the percentages of abnormal responses were 76% for ACS, 68% for BCV AFz and 56% for BCV at the mastoid. This could be explained by the difference in the percentage of NR to ACS for oVEMPs between the two groups of patients (41% of the 63 patients versus 0% of the 37 patients).

**Figure 3:**
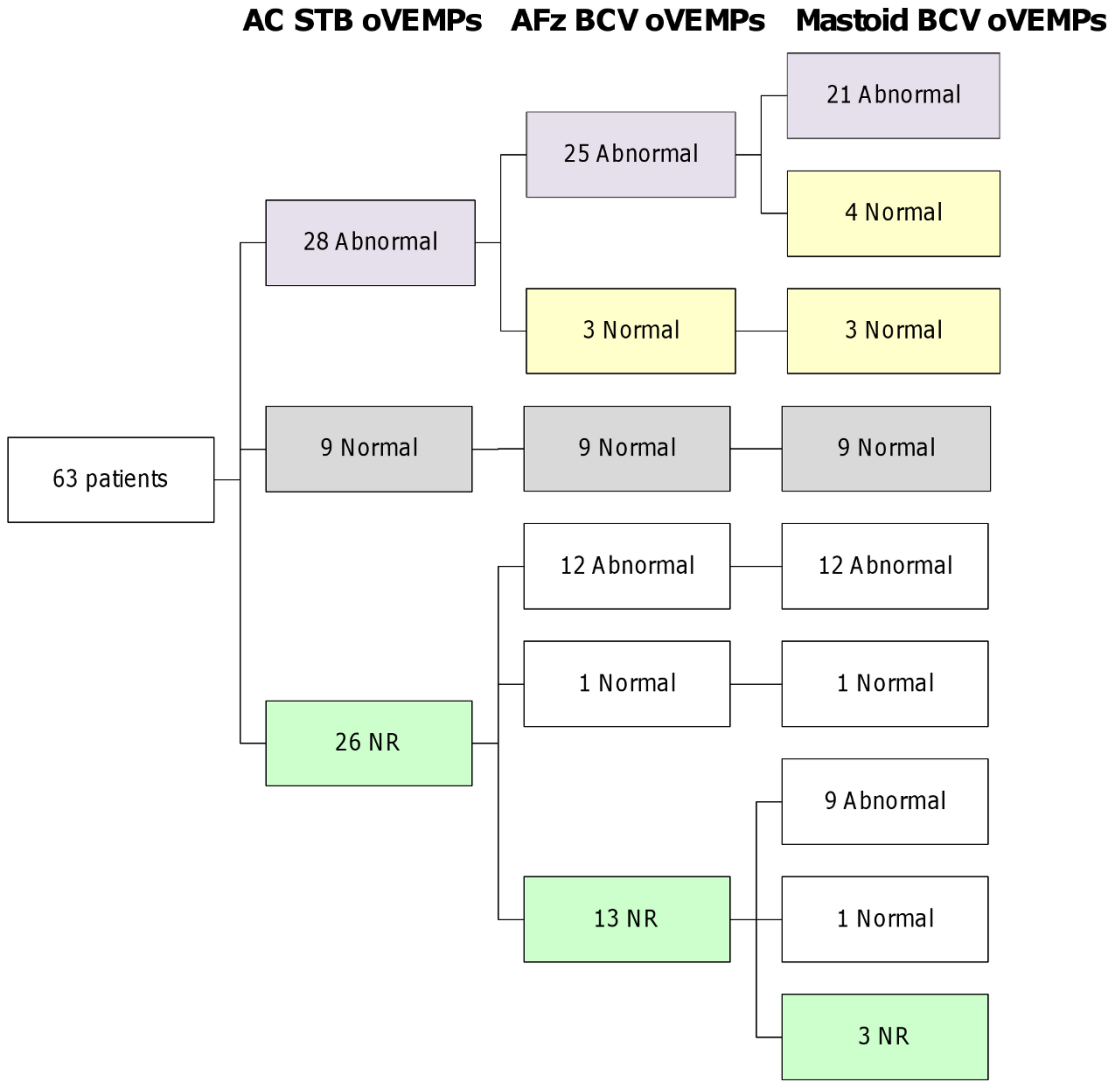
Flowchart showing the oVEMP results for non-operated VS patients (n = 63). Results are presented according to the following test sequence: AC STB, AFz BCV and mastoid BCV. This sequence is optimal to provide reliable conclusions about the functional status of the inferior (mostly utricular) nerve in a minimum of time. Patients with normal ACS oVEMPs always exhibited normal AFz and normal mastoid oVEMPs (Figure 3, grey squares). Patients with abnormal oVEMPs also exhibited abnormal AFz (25 of 28 patients) and abnormal mastoid oVEMPs (21 of 28 patients) (Figure 3, purple squares). Abnormal ACS but normal BCV oVEMPs were observed in three of 28 patients for AFz and in seven of 28 patients for mastoid BCV (Figure 3, yellow squares). Among the 26 ACS NR patients, 13 were NR for AFz BCV and three were NR for mastoid BCV(green squares).

Dysfunction of the superior vestibular nerve was detected almost equally with ACS (75.7%) and with AFz BCV (67.6%) stimulation in the VS patients with positive response from the unaffected side to ACS. Most of the time, ACS and AFz BCV gave similar results regarding the function of the superior vestibular nerve. All patients with normal ACS oVEMPs exhibited normal AFz and normal mastoid oVEMPs (Figure 3, grey squares). On the other hand, patients with abnormal ACS oVEMPs also exhibited abnormal AFz BCV oVEMPs (in 89%: 25/28 patients; Figure 3, purple squares). However, in some patients (11%, 3 of 28), we noticed some dissociation between the two modes of stimulation: abnormal ACS but normal AFz oVEMPs.

We recommend that both ACS and AFz BCV stimulations are necessary in VS patients to screen superior vestibular nerve function. In most cases, they gave similar findings. In a few patients, this was not the case. How can we explain this discrepancy?

1. It cannot result from a dysfunction of the middle ear because the findings of all the audiometric tests were normal such that even mild pathology of the middle ear could be ruled out.

2. ACS and BCV stimulations may stimulate different fibers of the superior vestibular nerve.

3. BCV may lead to false negative results, because, relative to ACS, it stimulates the superior vestibular nerve more strongly and possibly recruits more afferent primary vestibular neurons of the superior vestibular nerve.

With mastoid BCV stimulation, dysfunction of the superior vestibular nerve was detected in only 57% in the VS patients with positive responses from the unaffected side to ACS. Most of the time, AFz and mastoid BCV gave similar results regarding function of the superior vestibular nerve. All patients with normal AFz oVEMPs exhibited normal mastoid oVEMPs (Figure 3, grey squares). On the other hand, patients with abnormal AFz oVEMPs also exhibited abnormal mastoid oVEMPs (in 84%: 21 of 25 patients) (Figure 3, purple squares). In some patients (16%, 4 of 25), we noticed some dissociation between the two modes of stimulation: abnormal AFz BCV but normal mastoid BCV oVEMPs.

However, these dissociations may result from the fact that mastoid stimulation is stronger than AFz stimulation, inducing a decrease oVEMP asymmetry and thus a false normal response [25]. Therefore, BCV at the mastoid should be used to assess the residual function of the superior vestibular nerve but mastoid BCV stimulation was not necessary when ACS and AFz gave results regarding the function of the superior vestibular nerve.

In patients with no oVEMP response on either side to ACS (26 patients), BCV was required to study the function of the superior vestibular nerve: 50% of these patients were responders with AFz (13/26 patients) and 88% with mastoid BCV (23/26 patients) stimulation (Figure 3, white squares). Abnormal function of the superior vestibular nerve could still be detected in NR patients to AFz.

The NR patients in our study were significantly older (63±12 years, t-test) than patients with positive responses from the intact side (51±12 years). This is consistent with reports in the literature, which indicate that the excitability of the superior vestibular nerve for BCV and ACS decreases with age [26]–[27]. Only oVEMPs evoked by forehead-taps and lateral accelerations were not influenced by the subjects' age [28]–[29].

### ACS and BCV oVEMPs: in which order should the stimulations be done?

We suggest that ACS stimulation should be performed first because of the observed sensitivity in responder patients, and its independence of the operator. In addition, ACS stimulation allows comparison in VS patients of the function of both superior and inferior nerves using the same mode of stimulation. BCV at AFz is a stronger stimulus but oVEMPs data depends on the position of the vibrator at AFz (operator-dependent) and on the skull anatomy (patient-dependent).

**Figure 4:**
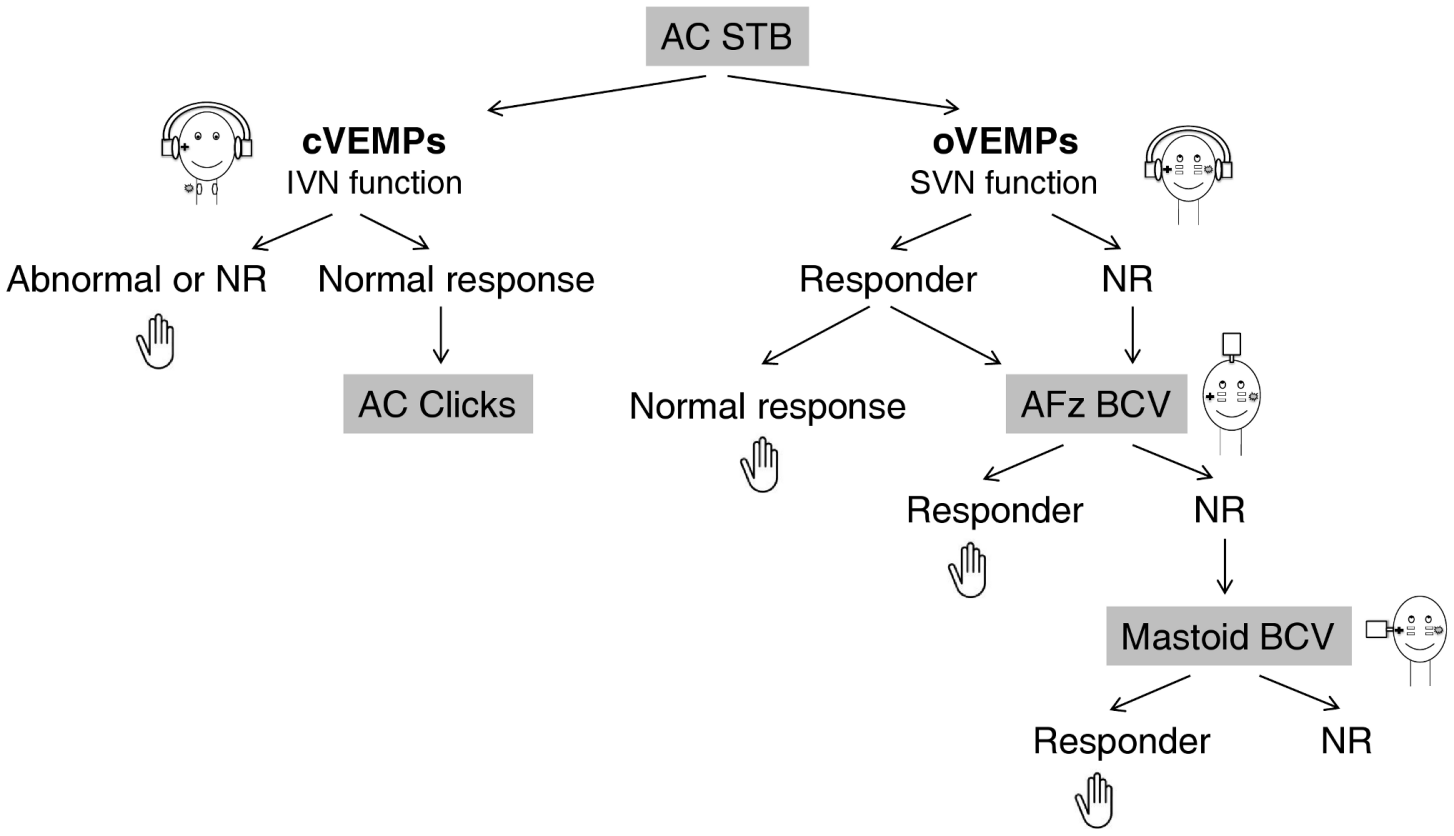
Flowchart illustrating in which order the stimulations (ACS and BCV) should be done to test predominantly the function of the superior and mostly of the utricular nerve using oVEMPs. IVN: inferior vestibular nerve - SVN: superior vestibular nerve. ACS stimulation may be performed first in patients suffering from vertigo. If ACS induced normal oVEMPs, BCV was not required. If ACS gave abnormal oVEMPs, bone stimulation should be done. We suggest beginning with AFz BCV stimulation: this mode stimulates both labyrinths equally and is easier to perform than mastoid stimulation in a routine clinical setting. When no response was observed on either side to ACS and to AFz stimulation, mastoid BCV stimulation is necessary to appreciate the residual function of the superior vestibular nerve.

If ACS induces normal oVEMPs, BCV is not required (Figure 4). If ACS gives abnormal oVEMPs, bone stimulation at AFz could be done to confirm the abnormal function of the superior vestibular nerve.

In contrast, BCV stimulation may be the only way to determine the superior vestibular nerve function in non-responder patients to ACS. We recommend beginning with AFz BCV stimulation: this mode stimulates both labyrinths equally [30] and is easier to perform than mastoid stimulation in a routine clinical setting. AFz and mastoid stimulation provide similar oVEMPs in most of the patients. Thus, when AFz stimulation is conclusive, mastoid BCV stimulation is not necessary (Figure 4).

Finally, when no response is observed on either side to ACS and to AFz stimulation, mastoid BCV stimulation is necessary: this stimulation may be the only approach to provide information about the superior function (Figure 4).

More generally, ACS and not BCV should be used in first instance in a prospective study to screen for the causes of the vestibular disease and to be sure that a superior canal dehiscence is not present [31].

**In conclusion**, both cervical and ocular VEMP tests contributed to assessing the vestibular profile of patients suffering from VS. In addition, in some VS patients, an abnormality of vestibular nerve function was only detected using VEMPs. For oVEMPs, we suggest that ACS stimulation should be used first. In patients who respond to ACS and who have normal responses, this gives enough information about the functional status of the superior vestibular nerve, and BCV stimulation is not required. In patients with abnormal responses on the affected side with ACS, it may be useful to perform BCV at AFz to confirm abnormal function of the superior nerve.

In patients with no responses on either side to ACS, BCV is the only approach allowing assessment of the function of the superior vestibular nerve. We favor using AFz BCV stimulation first because of it is easier to perform in routine clinical practice than mastoid stimulation. Mastoid stimulation is useful in patients who are non-responders to both ACS and AFz BCV stimulations.
